# Emerging mental health problems during the COVID-19 pandemic among presumably resilient youth -a 9-month follow-up

**DOI:** 10.1186/s12888-021-03650-z

**Published:** 2022-01-27

**Authors:** Stine Lehmann, Jens Christoffer Skogen, Gro M. Sandal, Ellen Haug, Ragnhild Bjørknes

**Affiliations:** 1 Department of Health Promotion and Development, Faculty of Psychology, The University of Bergen, Postboks 7807, 5020 Bergen, Norway; 2 Department of Health Promotion, Norwegian Institute of Public Health, Bergen, Norway; 3 Alcohol & Drug Research Western Norway, Stavanger University Hospital, Stavanger, Norway; 4 Department of Public Health, Faculty of Health Sciences, University of Stavanger, Stavanger, Norway; 5 Department of Psychosocial Science, Faculty of Psychology, The University of Bergen, Bergen, Norway; 6 Department of Teacher Education, NLA University College, Bergen, Norway

## Abstract

**Background:**

The COVID-19 pandemic may have multifarious adverse effects on the mental health of some youth. To our knowledge, no study has followed young people beyond the first 6 months of the pandemic outbreak. The aim of this study was to examine 1) Change in internalizing, externalizing, and total mental health problems over two time-points with a nine-month interval during the COVID-19 outbreak and 2) Whether contextual and COVID-19-related factors contribute to change in mental health problems.

**Methods:**

Youth within the municipality of Bergen aged 11-19 years were invited via SMS to participate in an online survey in April and again in December 2020. A total of 2997 (40% response rate) youth participated at baseline in the present study, and 1598 (53.3%) completed the second survey. At baseline, the mean age was 16.0 (standard deviations 1.7) years, about 60% were girls, and 93% were born in Norway. Comparison across time was approached using inferential statistics and mixed linear models with maximum likelihood estimation and mixed-effects logistic regression models.

**Results:**

There was an overall increase in total mental health problems from the first weeks into lockdown to 9 months after the pandemic outbreak. The overall increase seems to be exclusively driven by internalizing difficulties, i.e., increases in emotional problems and peer problems. The level of externalizing difficulties, i.e., conduct problems and hyperactivity/inattention remained stable between the two time-points.

**Conclusions:**

Our results imply that in the wake of the pandemic, one should be aware of emerging mental health problems among presumably resilient youth, in addition to the more expected and pronounced mental health needs of vulnerable groups. Efforts to reach out to the general youth population with preventive measures in schools may be important actions to normalize the situation for young people, and to identify those in need of more targeted mental health interventions.

## Background

The Covid-19 pandemic and the consequential disease-suppressive measures have made their distinct mark on the years 2020 and 2021 for young people. Social distancing and worries about short-term medical crises and long-term consequences represent critical stress factors on youth mental health [1–3]. Moreover, young people seem to be particularly vulnerable to the impact of measures on their mental health compared to older age groups [4, 5]. Even so, it is unclear to which degree and in what way the disease-suppressive measures are affecting young people’s mental health in the short and long term [6, 7]. In this study, we investigate changes in youth mental health problems during the Covid-19 pandemic from April to December 2020 in Norway.

On March 12th, Norwegian authorities announced a national lockdown to suppress the spread of the Covid-19 infection [8]. For young people, digital home-schooling and the closing of organized leisure activities were among the measures most immediately affecting their everyday life. In spring 2020, these national measures lasted for 3 months, with a reopening of schools in mid-June. During a brief period before and after summer vacation, schooldays returned to normal, only interrupted by local outbreaks requiring the single schools or classes to be quarantined. In November, however, schools were closed again in the municipality of Bergen after the second wave of outbreak. Overall, from March to December 2020, a repeated combination of national, municipal, and local measures caused interferences in everyday life of youth, with a decrease and unpredictability in social contact, physical school attendance, and access to leisure activities.

To date, few studies have used a longitudinal design to explore stability and change in youth mental health problems during the first year of the pandemic. Among these, results have been mixed. A US study showed increased parent-assessed youth mental health problems from before the outbreak, after controlling for changes associated with maturation [9]. This study leaves it unclear what type of symptoms were increasing. Prospective longitudinal studies have demonstrated deterioration in mental health among youths during the pandemic. Among young people in Australia, anxiety, and depression symptoms increased 2 months into the stay-at-home government directive, including online learning for students compared to before the outbreak [2]. In this study, female sex, COVID-19 related worries, online learning difficulties, and increased conflict with parents predicted increase in mental health problems, whereas adherence to stay-at-home orders and feeling socially connected during the lockdown protected against poor mental health. In a prospective Norwegian study of 13-16 year old’s [10], clinical levels of mental health problems increased from 5.3% in 2019 to 6.2% in June 2020. Female sex, pre-pandemic mental health problems, and a single-parent household predicted more mental health problems in June 2020. Somewhat surprisingly, youth from low-income families or a history of maltreatment showed significantly less increase in mental health problems compared with peers who did not report such difficulties. However, this study only measured the change in internalizing problems during the pandemic. The possible impact of the lockdown on externalizing problems and total symptom-load across symptom dimensions is still unknown in a Norwegian context and is scarce internationally.

In sum, results from prospective longitudinal studies indicate an increase in mental health problems from pre-pandemic levels. However, studies following young people’s mental health over a longer period with more than two waves of data yield a somewhat different picture. A US study following youths between March and July 2020 found that symptoms of depression and anxiety peaked around late April/early May and then decreased through May-July [11]. This is in line with another US study of youth, reporting an increase in depression, anxiety, and oppositional/defiant symptoms from pre-COVID-19 to spring 2020; followed by a decrease during summer 2020 [12]. Here, lower family income was related to increases in inattention, whereas higher family income was related to increased oppositional/defiant symptoms.

Taken together, these results show that mental health problems among youths may change in different manners over the course of the pandemic depending on individual and contextual factors, like increasing age, female sex, low socioeconomic status, and social connectedness. Further, recent findings suggest increased mental health problems among adult immigrants compared to non-immigrants during the COVID-19 pandemic [13]. Surprisingly, few studies conducted during the pandemic have included these variables as possible contributors to stability and change in mental health among youth [2]. We argue that these potential risk factors might operate differently during the pandemic as restriction measures impact population groups differently. Also, studies report that youth experience isolation and loneliness because of the restriction measures [14–17]. Youth’ feelings of loneliness over the course of the pandemic, and associated characteristics, warrant more research attention given the implications of loneliness for mental health [1]. To date studies investigating the impact of loneliness on youth mental health during the COVID-19 pandemic seem sparse. Sleep problems are repeatedly shown to be strongly associated with internalizing problems among youth [18]. Furthermore, results from the first wave of data (T1) in the current study, showed that 19% of the participating youth reported increased sleep problems after schools closed in March 2020 [19]. Hence sleep problems is an important factor to control for when studying change in internalizing problems over time during the Covid-19 pandemic.

Overall, studies suggest, to a varying degree, that the COVID-19 pandemic may have multifarious adverse effects on the mental health for some youth. We advocate that additional studies are needed about the pandemic’s long-term effects that include complex set of possible predictors. To our knowledge, no study has followed young people over a longer period of the pandemic outbreak stretching beyond the first 6 months.

Against this background, the aim of this study was to examine the impact of the Covid-19 pandemic and disease-suppressive measures on young people’s mental health. More specifically, in a general sample of youth aged 11-19 years, we examine: 1) Change in internalizing, externalizing, and total mental health problems over two time-points with a nine-month interval, and 2) Whether contextual and COVID-19-related factors contribute to change in mental health problems.

## Methods

### Design and setting

The study *COVID-19 Young* is a longitudinal study of young people aged 11-19 attending secondary and high schools within the municipality of Bergen, Norway [19]. The data collection so far comprises two waves. The first data collection (t_1_) started 27th of April 2020, during the 7th week of the national lockdown, and closed on the 11th of May. The second wave of data (t_2_) was collected between the 16th of December 2020 and 10th of January 2021, during local restrictions implying partly closed schools and sports- and leisure activities put on hold.

The study comprises two subsamples: Cohort 1 were young people aged 12-15 years whose parents participated in the Bergen in Change study [20], where a random sample of 81,170 individuals from a total of 224,000 adult inhabitants (aged 18–99 years) in the city of Bergen, Western Norway, were invited to participate. Parents in this study consented to their child (ren) participating in the present study. Upon consent, parents provided contact information for the youth. A total of 1565 youth were contacted in cohort 1 in wave 1. The consenting parents were more often females (Cramérs V: 0.069, *p* < 0.001), older (Cramérs V: 0.092, *p* < 0.001), had higher educational attainment (Cramérs V: 0.155, *p* < 0.001) and household income (Cohen’s D: 0.19, p < 0.001), and had less often shared residence for the child (Cramérs V: − 0.054, *p* = 0.006) when compared to non-consenting parents [19]. These differences were in the range between very small and small effect sizes. Cohort 2 were young people aged 16-19 years, attending high school. Following Norwegian legislation, young people 16 years and older consent on their own behalf. For this cohort, the county council provided phone numbers from their school contact registers. All young people registered here were invited to participate A total of 5947 youth was contacted in Cohort 2 in wave 1.

The invitation procedures were the same for cohorts 1 and 2 in both waves of data collection. Youth were recruited via SMS and a link to a secure online platform containing an information letter and a 15–30-min survey. Two SMS reminders were sent. Participants provided informed concent to participate by ticking a consentform at the start of the survey. In both waves participants were included in a lottery for a new cellphone as an incentive to participate.

### Characteristics of participants

In wave 1, a total of 7512 youth was invited to participate. Of these, 843 (54%) in cohort 1 and 2154 (36%) in cohort 2 responded, yielding a total of 2997 (40%) youths completing the T1 survey. The mean age was 16 years (SD 1.7), 57.7% were females, and most participants reported living with both parents (77.5%), being born in Norway (93%), and living with siblings (71%). All participants from wave one was invited to answer the second survey. A total of 1598 (53.3%) young people completed the second survey.

### Measures

#### Predictor

The predictor in the present study was a variable differentiating between t_1_ and t_2_.

#### Covariates measured at baseline

The included demographic covariates were self-reported age, gender, and country of birth. Age was reported in whole years, and gender differentiated between “boy” and “girl”. We differentiated between being born in Norway and being born in another country. Additional covariates included questions about experiencing loneliness, home school learning, perceived situation at home with one’s family, sleep-problems, and nightmares.

*Loneliness* was gauged using the single question “Have you felt lonely?” with five possible response options Never (=0), Seldom (=1), Somewhat often (=2), Very often (=3) and Always (=4). For the purposes of the present study, we differentiated between Seldom or less (=0) and Pretty often or more (=1).

Regarding *home school learning* the participants were asked “During the weeks of home-schooling, do you feel that you have learned …” where the response options were Less (=2), About the same (=1) and More (=0). For *situation at home*, the participants were asked “How are you getting along with your family during this period after school closing?”, where the response options were A lot better (=1), A little better (=2), As before the school closed (=3), A little worse (=4) and A lot worse (=5). In the present study, we differentiate between Better (=1), The same (=2), and Worse (=3).

For *sleep problems*, we assessed difficulties initiating and maintaining sleep (DIMS) with the following question “During the last couple of weeks, after the closing of your school: Have you had problems sleeping or do you wake up frequently during the night?” with three response options “not true” (=1), “somewhat true” (=2), “true” (=3). *Nightmares* were assessed using the question “After the schools closed, have you had nightmares or unpleasant dreams?” with three options “Yes, more often than before” (=1), “Not more than before” (=2), “I do not have nightmares or unpleasant dreams” (=3). The phrasing of all items may be found in supplementary Table 1 in Lehmann et al. 2021 [19].

#### Mental health

Mental health was measured by the 25-item Strengths and Difficulties Questionnaire (SDQ, [21]). The SDQ is a mental health questionnaire for 3- to 17-year-olds. A self-report version of the SDQ is available for young people aged 11-17 years. SDQ comprises five subscales: emotional problems; conduct problems; hyperactivity; peer problems; and prosocial. Each subscale contains five items rated on a three-point-scale (0-1-2), yielding a subscale-score in the range of 0–10. The scores (excluding the scale for prosocial behaviors) can be combined into a total difficulties score, with scores ranging between 0 and 40. In addition, the emotional problems and peer problems subscales can be combined to comprise the intermediate subscale “internalizing problems”, while conduct problems and hyperactivity subscales can be combined to comprise “externalizing problems”. For the purposes of the present study, we present the total scale scores as well as the individual subscales scores and the intermediate subscale scores Internalizing and Externalizing, at the two time points. In analyses including covariates we only present internalizing and externalizing problems. Cut-off points are available for self-completed SDQ for the total scale and the subscales (SDQEnglishUK4-17scoring-1.PDF (ehcap.co.uk). In the present study we used recommended cut-points to differentiate between “close to average” and “slightly raised (/slightly lowered)” versus “high (/low)” and “very high (/very low)”.

### Statistical analyses

First, baseline characteristics for those with valid scores on at least one SDQ subscale at baseline were calculated. Next, the valid observations, mean scores, and standard deviations across time points of the SDQ total scale and subscales were computed.

Comparison of SDQ scores across time was approached using inferential statistics: Initially, a series of paired t-tests were computed for SDQ total and all subscales, followed by estimating the same associations using mixed models. Mixed models are statistical models that contain both fixed and random effects. In longitudinal analyses, mixed models hold the advantage that they make use of all available data points. For the continuous SDQ-measures mixed linear models with maximum likelihood estimation were employed, while mixed effects logistic regression models were used for the dichotomous SDQ-measures. The impact of the included covariates was investigated using the SDQ internalizing and the SDQ externalizing subscales only. Separate adjustment for each covariate is presented across the two subscales, as well as the fully adjusted models. Finally, we investigated the potential moderating role of the covariates in a series of interaction models, and statistically significant interactions were presented in stratified analyses. In order to make full use of the available data, pair-wise deletion was employed for handling of missing data. Valid responses on the variables included in the regression models ranged from *N* = 2678 (100%) in the crude model to 2530 (94.5%) in the fully adjusted model at baseline. As we employed mixed models, we were able to use the full data set, including information from those participants who only participated at t_1_. We assessed attrition at t_2_ by comparing age, gender and country of birth among the individuals that participated at t_1_ only with the individuals who participated at both time-points.

#### Post-hoc analysis

To avoid tautological interpretation of the apparent moderating effect of loneliness on the SDQ internalizing subscale, we also estimated interaction models for SDQ Emotional Problems and SDQ Peer Problems separately as a post-hoc analysis.

## Results

### Sample characteristics

A total of *N* = 2997 participated at baseline in the present study. Out of the total number of participants, *n* = 2678 had valid scores on at least one SDQ subscale at baseline and were retained for further analyses. At baseline, the mean age of the study sample was 16.0 (1.7 standard deviations) years, and about 59% were girls, and 93% were born in Norway. At follow-up, these numbers were similar, with a mean age of 15.9 (1.7 standard deviations), 61% were girls, and 94% were born in Norway. Those participating at both time-points were marginally younger compared to the t_1_-only participants (mean age 15.9 years vs 16.1, *p* = 0.001). Girls were slightly more likely to participate at both time-points compared to boys (57.0% girls among the t1-only participants vs 62.2% girls participating at both time-points, *p* = 0.032). Country of birth was not associated with attrition at t_2_ (*p* = 0.356). An overview of the SDQ total scale and subscales is presented in Table 1, including valid observations, mean and standard deviations across the two time-points.

**Table 1:** Mean scores of SDQ total and subscales at t_1_ and t_2_

	t1: 6-7 weeks into lockdown			t2: 9-month follow-up		
	Valid obs	M	SD	Valid obs	M	SD
SDQ Total difficulties	2617	11.3	5.3	1071	11.6	5.7
SDQ Emotional symptoms	2676	3.3	2.5	1105	3.9	2.7
SDQ Peer problems	2676	2.1	1.7	1106	2.3	1.7
SDQ Inattention-hyperactivity	2678	4.4	2.2	1104	4.1	2.3
SDQ Conduct problems	2677	1.5	1.4	1105	1.4	1.4
SDQ Prosocial behavior	2677	7.9	1.6	1107	8.0	1.7
SDQ Internalizing problems	2632	5.4	3.4	1078	6.2	3.7
SDQ Externalizing problems	2635	5.9	3.1	1078	5.5	3.0

### Comparison of mental health across time points

Increased mean scores from t_1_ to t_2_ were observed for SDQ total scale, driven by an increase in the internalizing subscales emotional symptoms and peer problems (Table 2). No statistical difference was observed for externalizing subscales or prosocial behavior.

**Table 2:** Paired t-tests of SDQ total and subscales at t_1_ and t_2_

	Valid obs	Mean t1	Mean t2	Difference t2-t1	*p*-value
SDQ Total difficulties	1052	11.1	11.7	0.6	**< 0.001**
SDQ Emotional symptoms	1104	3.4	3.9	0.5	**< 0.001**
SDQ Peer problems	1105	2.2	2.3	0.1	**0.002**
SDQ Inattention-hyperactivity	1104	4.1	4.1	0.0	0.996
SDQ Conduct problems	1104	1.4	1.4	0.0	0.674
SDQ Prosocial behavior	1107	7.9	8.0	0.0	0.310
SDQ Internalizing problems	1064	5.6	6.2	0.6	**< 0.001**
SDQ Externalizing problems	1067	5.5	5.5	0.0	0.839

Bold indicates statistical difference at alpha< 0.05

The results from the mixed models yielded similar results as the paired t-tests (Table 3). The mean score of the SDQ total scale increased from t_1_ to t_2_, driven by the internalizing subscale, while no statistically significant change was observed for the externalizing subscale or prosocial behavior subscale. Using cut-points, the increase observed in the internalizing subscales corresponded to increased odds of 2.18 for emotional symptoms and 1.55 for peer problems subscale.

**Table 3:** Comparison of SDQ total and subscales at t_1_ and t_2_. Results from mixed models

Subscale	Coefficient^a^	Odds ratio^b^
SDQ Total difficulties	0.501*** (0.123)	1.44*
SDQ Emotional symptoms	0.532*** (0.055)	2.18***
SDQ Peer problems	0.162*** (0.042)	1.55**
SDQ Inattention-hyperactivity	−0.090 (0.054)	1.00
SDQ Conduct problems	−0.034 (0.036)	1.18
SDQ Prosocial behavior	0.072 (0.042)	1.19
SDQ Internalizing problems	0.650*** (0.079)	NA
SDQ Externalizing problems	−0.136 (0.071)	NA

*NOTE:* Standard errors are in parentheses

*** *p* < 0.001, ** *p* < 0.01, * *p* < 0.05; ^a^Mixed linear models; ^b^Mixed effects logistic regression models; NA: Cut-offs not applicable SDQ internalizing and SDQ externalizing problems. Bold indicates statistical difference at alpha< 0.05

Table 4 shows the change in internalizing and externalizing coefficients, adjusted for covariates. First, we estimated the association between time and the two subscales when including covariates separately, and then in a fully adjusted model. For both subscales, the inclusion of covariates changed the point estimates only marginally across models. For externalizing problems, separate adjustment for lonely and situation at home increased the point estimates slightly and in both cases the association became statistically significant.

**Table 4:** SDQ internalizing and SDQ externalizing problems. Crude versus adjusted estimates. Separate adjustments, and fully adjusted. All covariates measured at baseline

	SDQ Internalizing	SDQ Externalizing
Crude	0.650***	−0.136
Adjusted for age	0.663***	−0.136
Adjusted for gender	0.637***	−0.130
Adjusted for birth country	0.653***	−0.137
Adjusted for lonely	0.658***	−0.148*
Adjusted for home school learning	0.649***	−0.133
Situation at home	0.659***	−0.145*
Adjusted DIMS	0.691***	−0.118
Adjusted Nightmares	0.661***	−0.120
Fully adjusted	0.671***	−0.115

Standard errors are in parentheses

*** *p* < 0.001, ** *p* < 0.01, * *p* < 0.05; Bold indicates statistical difference at alpha< 0.05

Next, we estimated the association with time for the internalizing and externalizing subscales when introducing covariates as an interaction term in the base model. Covariates were included one at a time. The main effects and interaction with time for the included covariates are given in Table 5. For the internalizing subscale, main effects were observed for all covariates (all *p* < 0.01), while there was no main effect of age (*p* = 0.077) and birth country (*p* = 0.842) in the mixed models for the externalizing subscale. Concerning interaction effects, loneliness and situation at home were statistically significant in the internalizing subscale models, and birth country, situation at home and sleep problem (DIMS) was statistically significant in the externalizing models.

**Table 5:** SDQ internalizing and SDQ externalizing problems, association with selected covariates. Main effect and interaction with time. All covariates measured at baseline

	SDQ Internalizing	SDQ Externalizing
Age	**< 0.001**	=0.077
Age×time	=0.688	=0.364
Gender	**< 0.001**	**=0.008**
Gender×time	=0.111	=0.155
Birth country	**=0.010**	=0.842
Birth country×time	=0.183	**=0.022**^a^
Lonely	**< 0.001**	**< 0.001**
Lonely×time	**=0.001**^a^	=0.735
Home school learning	**=0.006**	**< 0.001**
Home school learning×time	=0.399	=0.148
Situation at home	**< 0.001**	**< 0.001**
Situation at home×time	**=0.024**^a^	**=0.017**^a^
DIMS	**< 0.001**	**< 0.001**
DIMS×time	=0.157	=**0.008**^a^
Nightmares	**< 0.001**	**< 0.001**
Nightmares×time	=0.774	=0.199

Bold indicates statistical difference at alpha< 0.05

^a^Retained for further stratified analyses

**Fig. 1:**
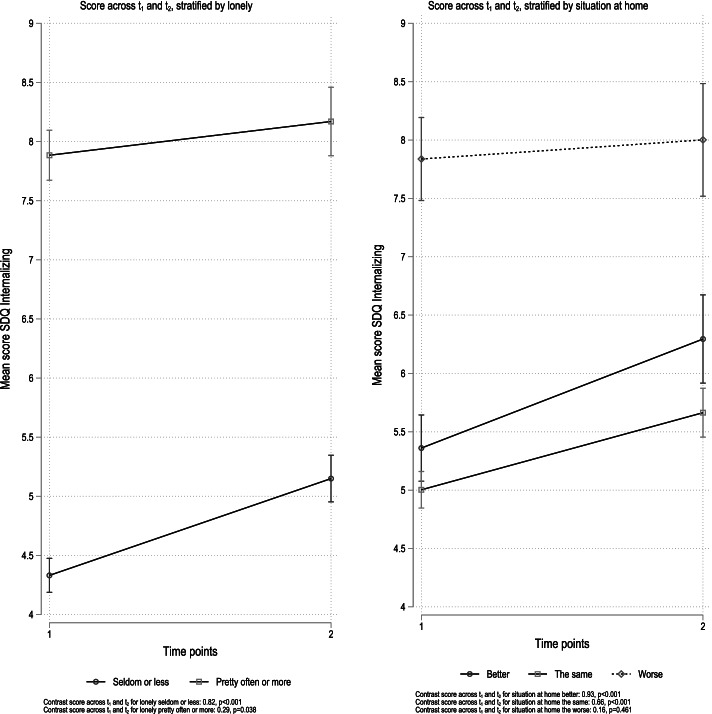
Results from stratified analyses. Internalizing sub-scale. Stratification variables loneliness and situation at home

**Fig. 2:**
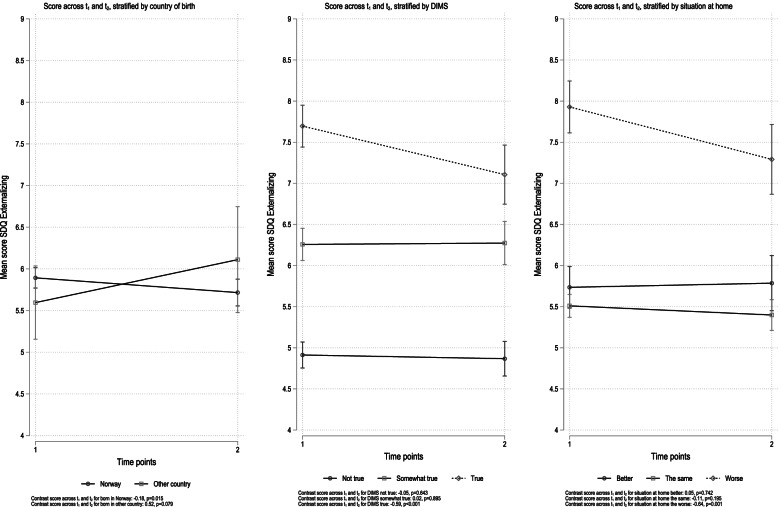
Results from stratified analyses. Externalizing sub-scales. Stratification variables country of birth, sleepproblems, situation at home

The results from stratified analyses for these covariates and corresponding SDQ subscales are given in Figs. 1 and 2. For the externalizing subscale, there was a significant decrease between time points in symptoms for the group born in Norway (coefficient − 0.18, *p* = 0.015) and a positive but non-significant increase for the group born in any other country (coefficient 0.52, *p* < 0.079) (Fig. 1). In the analyses stratified for sleep problems, there was a significant decrease in externalizing symptoms among those who reported sleep problems (“true”: coefficient − 0.59, *p* < 0.001), but not for those who responded, “not true” (coefficient − 0.05, *p* = 0.643) or “somewhat true” (coefficient 0.02, *p* = 0.895) (Fig. 1). For externalizing problems stratified for situation at home, there was no change in problems across time points for those who reported the situation at home being better (coefficient 0.05, *p* = 0.742) or the same as before (coefficient − 0.11, *p* = 0.195) (Fig. 2). There was, however, a significant decrease in externalizing problems among those who reported the situation at home as worse (coefficient − 0.64, *p* = 0.001).

Regarding internalizing problems, both those who reported being lonely “seldom or less” and “pretty often or more” reported more problems across time points (Fig. 1). The increase in internalizing problems was, however, larger among those reporting being lonely seldom or less (coefficient 0.82, *p* < 0.001) compared to those who reported being lonely pretty often or more (coefficient 0.29, *p* = 0.038). For internalizing problems stratified for situation at home, there was an increase in problems across time points for those who reported the situation at home being better (coefficient 0.93, *p* < 0.001) or the same as before schools closed (coefficient 0.66, *p* < 0.001), but not for those who reported the situation as worse (coefficient 0.16, *p* = 0.461) (Fig. 2).

**Fig. 3:**
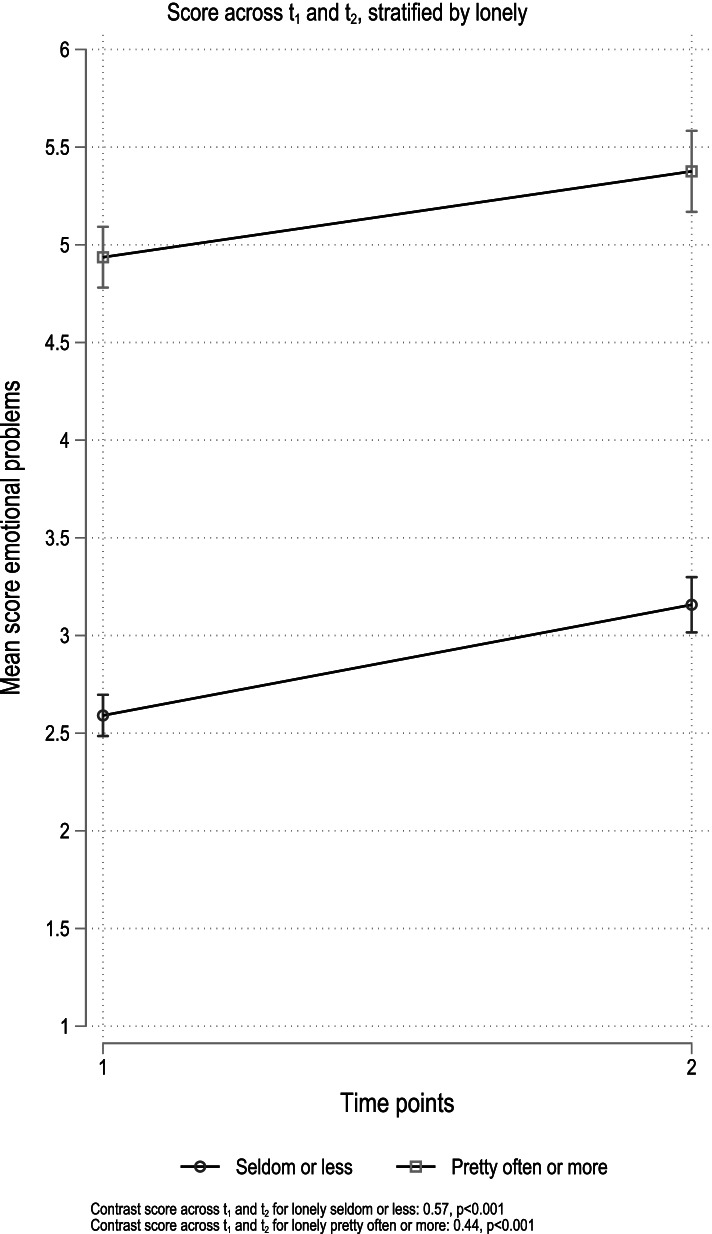
Results from stratified analyses. Emotional problems. Stratification variable loneliness

**Fig. 4:**
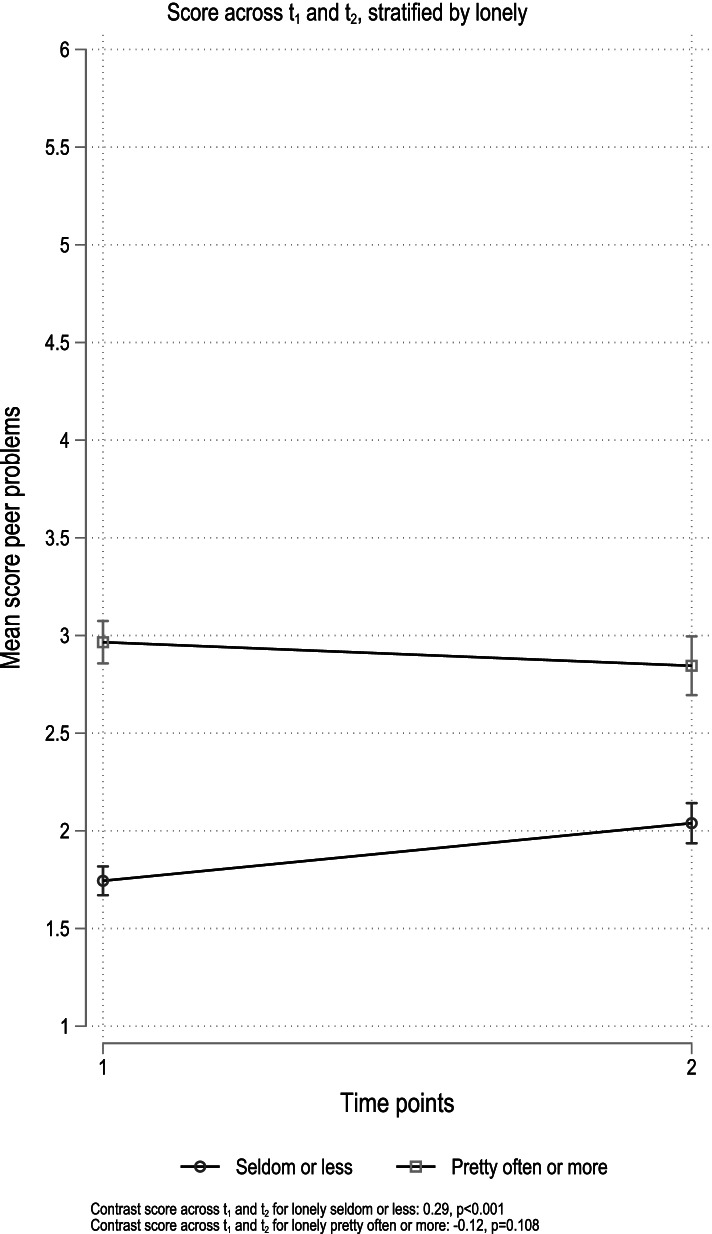
Results from stratified analyses. Peer problems. Stratification variable loneliness

In the post-hoc analysis, there was a significant increase in emotional problems regardless of level of loneliness at baseline, and no evidence for an interaction (*p* = 0.274; Fig. 3). There was, however, a significant interaction between being lonely and time for peer problems (*p* < 0.001; Fig. 4). For those who reported being lonely seldom or less, we observed an increase in reported peer problems across time points (coefficient 0.29, *p* < 0.001), while no change was observed among those who reported being lonely pretty often or more at baseline (coefficient − 0.12, *p* = 0.108).

## Discussion

This study shows an overall increase in mental health problems from the first weeks into lockdown to 9 months after the pandemic outbreak. The overall increase seems to be exclusively driven by internalizing difficulties, i.e., increases in emotional problems and peer problems. The level of conduct problems and hyperactivity/inattention remained stable between the two time-points. Finally, the participating youths reported no change in prosocial behavior from the first to the second wave of data collection.

Our main result, an increase in internalizing symptoms, is in line with two other longitudinal studies, identifying increased internalizing problems from pre-pandemic levels during the spring 2020 [9, 10]. Our findings add to the previous results from studies demonstrating a decrease of symptoms during the spring-summer of 2020 [11, 12], indicating a “normalization” period after the first adjustment. One may speculate that these studies demonstrating a decrease in mental health problems were influenced by summer leave and ease in national and local preventive measures during the summer-months. Our study, with 9 months between the two waves of data collection, demonstrate the need for longer-term follow up of the mental health among young people in the Covid-19 period. With only two observations we do not know when the internalizing symptoms increased among the youth, thus, interpretation should be made with caution. However, it is likely that the increase in internalizing symptoms found 9 months after the initial lockdown could partly be the cumulative effect of the adverse changes the pandemic and accompanying restrictions have had on the lives of young people.

The increase in internalizing symptoms across the two timepoints was minimally affected after the inclusion of potentially pertinent covariates. The results from the interaction analysis, however, somewhat nuanced this overall finding. Both those reporting being lonely “seldom or less” and “pretty often or more” reported more internalizing problems across time points (Fig. 1). The increase was, however, larger among those reporting being lonely seldom or less (coefficient 0.82, *p* < 0.001) compared to those who reported being lonely “pretty often or more” (coefficient 0.29, *p* = 0.038), even though the lonelier group reported overall more internalizing problems at both time-points, compared to the less lonely. A review of the impact of loneliness and disease containment measures on mental health in children and youths, across different pandemic outbreaks from the 1940s and upwards, concluded that social isolation and loneliness increase the risk of depression and anxiety both at the time at which loneliness was measured and as late as 9 years after [1]. The study further suggested that the duration of loneliness correlated more strongly with mental health problems than the intensity of the feeling of loneliness. This may explain our results, in that the experience of loneliness might have increased more for the less lonely group in early spring, during the nine following months, due to the ongoing pandemic suppressive measures imposed on youth between the time points. In line with this understanding, post-hoc analysis showed increased peer problems across time points (coefficient 0.29, *p* < 0.001) for those who reported being lonely seldom or less. No change was observed among those who reported being lonely pretty often or more at baseline (coefficient − 0.12, *p* = 0.108; Fig. 4).

We see a somewhat similar picture regarding the impact of the youth’s situation at home. There was an increase in internalizing problems across time points for those who reported the situation at home being better (coefficient 0.93, p < 0.001) or the same as before (coefficient 0.66, p < 0.001) but not for those who reported the home- situation as worse (coefficient 0.16, *p* = 0.461) (Fig. 2). The level of internalizing symptoms was higher for those reporting a worsening of their home situation, at both time-points. Taken together, it seems that the increase in internalizing symptoms from early to later in the pandemic, was greater for those of the youths that in the beginning reported being rather well-adjusted, both socially and in their close family.

There were however some noteworthy nuances regarding a decrease in externalizing symptoms between the two time points (coefficient - 0.036, *p* < 0.1; Fig. 3). A decrease in externalizing behavior was found in the group born in Norway (coefficient − 0.18, *p* = 0.015; Fig. 1). This was not found for youth born outside of Norway. Notably, youth who reported being lonely directly after the lockdown in April, had a decrease in externalizing symptoms 9 month after. The same decrease in externalizing problems was found for youth reporting that their situation in their family had become worse since the schools closed (coefficient − 0.64, *p* = 0.001).

Our findings indicate that for some youths, externalizing behavior is diminishing during the pandemic. As others has pointed out [22], the reduction in social and school responsibilities might be experienced as alleviating for some youth, contributing to explanaining of the decrease in externalizing behavior. Even so, our results are contradictory compared to previous studies from the US during the COVID-19 lockdown [8], concluding with an increased risk for elevated externalizing problems among youths with ADHD and poor emotion regulation abilities, during spring and summer 2020. With regards to our findings, future data collection in this ongoing longitudinal study might provide insights into whether the decrease in externalizing problems among youth born in Norway, and youth reporting loneliness is related to youth development, school obligations, family situation, or pandemic-specific issues.

### Strengths and limitations

A major strength of this study is its longitudinal design, where a large sample of young people has been followed 9 months into the pandemic. Still, there was no comparison condition. Because of this, it is not possible to ascribe any changes in youth mental health, specifically to the effects of COVID-19. Likewise, we do not have a pre-pandemic status for this sample. We can therefore not state whether the first wave of data represents a change of mental health from a normal situation. This has implications for our interpretation of the degree of change between T1 and T2. However, findings from studies on the adult population in Norway [23] and the UK [24] indicate an overall stability in mental health among adults during the first 6 months of the pandemic.

Similarly, mental health problems and COVID-19 related covariates were assessed at the same time at baseline (t_1_), so while it is possible to ascertain that these factors were associated with increases in mental health problems, the direction of those associations cannot be concluded. Further, this study relies on self-reported mental health at both time-points, thus might be subject to report bias.

Finally, considering the generalizability of our findings, one should be aware that the infection rates in Norway in this phase of the pandemic were considerably lower than in many other countries. Also, the sample is recruited from a demographically restricted area, even if it is Norway’s second-largest city. On the other hand, given regional variations in suppressive measures, our restricted sampling area allowed studying youth mental health where all participants were under similar conditions. One should note, however, that our sample was predominantly a selected group of youths that either had parents with higher educational level and household income compared to non-consenting parents. Also, all participants from the age range of secondary school were recruited through contact information from the school offices. Hence, we had no access to older youth who are not attending school. However, in Norway, 93% of the youth attends high school.

The severity of government-imposed restrictions differed widely based on rates of infection, especially during the second data collection. In the geographic area of our sample (Bergen), all schools were closed in April 2020 (t_1_) and most schools were closed in December (t_2_). This is representative of the situation for many youths in Europe during the pandemic. Still, in Norway, few municipalities had infection rates as high as Bergen in December 2020. Consequently, participants in this study were somewhat more affected by decease-suppressive measures than young people in general in Norway.

## Conclusions

The impact of desease-suppressive measures on young people’s mental health has been of great concern to policymakers, health- and social services and researchers. Our findings nuance the picture from the Norwegian national lockdown in April 2020 [19], in that the results show an overall increase in total mental health problems from the first weeks into the pandemic lockdown to 9 months after. It seems that the relative increase in internalizing problems was more marked among the presumably least vulnerable youth. Even though we still identify a high-risk group with comparably high levels of mental health problems, also reporting more loneliness and demanding family relations, the situation for this group seemed more stable and unrelated to the duration of the pandemic. This may imply that in the wake of the pandemic, one should be aware also of emerging mental health problems among presumably resilient youth in addition to the more expected and pronounced mental health needs of vulnerable groups. Efforts to reach out to the general youth population with preventive measures in schools may be important actions to normalize the situation for young people, and to identify those in need of more targeted mental health interventions.

## Data Availability

The datasets generated and/or analyzed during the current study are not publicly available due to national regulations on health research but are available from the corresponding author on reasonable request.
